# Pediatric Cardiac Surgery in Ethiopia: A Single Center Experience in a Developing Country

**DOI:** 10.4314/ejhs.v33i1.10

**Published:** 2023-01

**Authors:** Yayehyirad Mekonen Ejigu, Hiwot Amare

**Affiliations:** 1 Children's Cardiac Center of Ethiopia; 2 Department of Internal Medicine, Jimma University, Jimma, Ethiopia

**Keywords:** Cardiac Surgery, Ethiopia, Sub-Saharan Africa, Ventricular Septal Defect, Patent ductus Arteriosus

## Abstract

**Background:**

In developing countries, the diagnosis of congenital heart diseases (CHD) is growing as the availability of echocardiography is increasing with most diagnoses made after birth. However, the access to pediatric surgery is still low and is mainly done by global surgical campaigns rather than local surgeons. Ethiopia has trained its local surgeons, and this is expected to improve the care of children with CHD. We aimed to evaluate the experience of local pediatric CHD surgery and its outcome in a single-center in Ethiopia.

**Methods:**

A hospital-based retrospective cohort study was done by including all patients with CHD and acquired heart disease in patients under the age of 18 operated at children's cardiac center in Addis Ababa Ethiopia. We set in-hospital mortality, 30-day mortality, and the prevalence of complications including major complications after cardiac surgery as the primary outcomes.

**Results:**

A total of 76 children were operated. The mean age at the time of diagnosis and surgery was 4 (±5) and 7 (±5) years, respectively. Forty-one (54%) were female. Ninety five percent of the 76 operated children were with the diagnoses of congenital heart diseases while the rest (5%) with acquired heart disease. Of those with congenital heart disease, Patent ductus arteriosus (PDA) accounted for (33.3%), Ventricular septal defect (VSD) for 29.5% and Atrial Septal Defect (ASD) for 10% and Tetralogy of Fallot (TOF) for 5%. According to the RACS-1 category, 26 (35.1%) were in category 1, 33 (44.6%) were in category 2, 15 (20.3%) were in category 3 and none of the children were in category 4 and 5. In-hospital mortality was 2.6% whereas there was no patient who died within 30 days after discharge. Operative mortality was 2.6%.

**Conclusions:**

Various types of lesions were treated in the hands of the local teams with VSD and PDA ligations as the commonest of all. The 30day mortality was within acceptable range and this outcome shows congenital and acquired heart diseases can be operated on in developing countries with good outcome despite the limited resources.

## Introduction

Global efforts to reduce neonatal and infant mortality rates have been underway with special emphasis on low- and middle-income countries (1). Specifically, the third Sustainable Development Goals focused on ending preventable death of children under five by 2030 (2). Even though there are variabilities in different parts of the world, under-five mortality rates mainly due to communicable diseases have decreased by increasing vaccinations (3), mother to child transmission of HIV (4) and improving diagnostic and treatment facilities mainly at primary health care units (5).

Congenital heart diseases (CHD) occurs in 11.89 per 1000 children (6). Due to increased availability of diagnostic facilities, the of diagnosis and treatment of CHD in children has improved in developed countries (7). This led to increased survival of children with CHD into adulthood (7).

In developing countries, the diagnosis of CHD is growing as the availability of echocardiography is increasing with most diagnoses made after birth (8). But CHD surgery is still not readily accessible in these countries (9, 10]). In developing countries, including Ethiopia, CHD surgery has historically been done mainly by surgeons from high-income countries participating in surgical missions [9]. More recently, there has been a focus on increasing long term partnerships that train local pediatric cardiac surgeons (10). As of 2021, there are five cardiac surgeons for the whole country and additional one in training. In addition, there are only 12 trained pediatric cardiologists in Ethiopia as a whole.

There were 78 cardiac centers or units which were performing regular open-heart operations in Africa and from these 22 were in Sub Saharan Africa, with only 2 in the Horn of Africa (11). The Children's Heart Fund of Ethiopia (CHFE), a Nongovernmental organization established in 1989 set out to save the lives Ethiopian children suffering from congenital and acquired heart disease. According to documents acquired from the center (12), since its inception in 1989G.C. until 2009 it has helped save the lives of 5600 children, by sending them for life saving surgery abroad. In 2009, CHFE pulled its pivotal accomplishments, establishing a cardiac unit in Addis Ababa, Ethiopia with the contribution of the public society, Addis Ababa University and Chain of Hope, a charity organization in UK. A data from the center states since its establishment, the lives of more 5000 children are saved by various visiting teams who operated on Ethiopian children locally. As of 2017 a complete local team that is able to perform both open heart surgery and catheter interventions is on the ground and doing varieties of interventions both on children and adults and cooperation with international colleagues is still ongoing through surgical and catheterization missions from different parts of the world.

Ethiopia trained local cardiac surgeons as well as other members of a complete cardiac surgery team, and this is expected to improve the care of children with congenital heart disease. Therefore, we aimed to assess the experience of local pediatric surgery and its outcome in a single-center in Ethiopia with patients included in the study operated by a single operator, a pediatric cardiac surgeon.

## Methods

This hospital-based retrospective cohort study was conducted at the Children's Cardiac Center of Ethiopia based in Addis Ababa on children operated between July 2019 and May 2021. All patients under the age of 18 having either congenital or acquired heart disease were included. Those with age ≥18 years were excluded.

Data was collected retrospectively from hard copy patient charts after patients were discharged from the hospital. These charts include data records from the outpatient follow up clinic, cardiology ward, ICU, operating room and the perfusion team. The data were first filled into a google Forms predetermined questionnaire by the pediatric cardiac surgeon.

The collected data included age, weight, gender, cardiac procedure type with Risk Adjustment for Congenital Heart Surgery-1 category (RACHS-1), cardiopulmonary bypass (CPB) time and aortic cross clamp (Aox) time, intensive care unit (ICU) stay, postoperative length of stay, duration of mechanical ventilation, 30-days mortality, in-hospital mortality and operative mortality, the later defined as 30-days mortality combined with in hospital mortality.

**Outcome measures**: We set in-hospital mortality, 30-day mortality, and the prevalence of complications including major complications after cardiac surgery as the primary outcomes. In-hospital mortality was defined as death of any cause during hospitalization for surgery until discharge. A 30-day mortality was defined when death occurred outside of the hospital but within 30 days after cardiac surgery performed. Complication was defined as a deviation from the expected outcome of cardiac surgery.

Data were described by mean, median or proportion after distribution of data was tested using histograms. To assess the correlates of cardiac surgery complications, univariate analysis was done using fisher's exact test or logistic regression. Variables were included in the final multivariable logistic regression if univariate analysis showed P value < 0.25. Data analysis was performed using STATA version 12.1, StataCorp LP, College Station, Texas, USA. P <0.05 was considered as statistically significant.

**Ethical considerations**: Ethical approval was obtained from the Children's Cardiac Center to publish the data used in this paper.

## Results

We included 76 children who had surgery, 41 (54%) of which were females. The mean age at the time of diagnosis was 4 (±4) years. The mean age at time of surgery was 7 (±5) years. At the time of surgery, none were newborns, 2 (3.3%) were infants, 16 (26.2%) were toddlers and the rest were above the age of 3. The mean age time elapsed from diagnosis until surgery was 3 (±2) years. The number of children with Cyanotic heart disease was 6 and the rest 70 had acyanotic heart disease (Table 1).

**Table 1:** Types of Cardiac lesion among children operated at the Children cardiac center, Addis Ababa, Ethiopia^1^ (number of patients=76)

Variable	Value
Age at diagnosis, years^2^	4 (±5)
Age at time of surgery, years^2^	7 (±5)
Female, n (%)	41 (54)
Syndrome, n (%)	3 (3.9)
Types of cardiac lesions, n (%)	
Patent ductus arteriosus	32 (33.3)
Ventricular septal defect	28 (29.5)
Atrial septal defect	10 (10.4)
Discrete Subaortic Stenosis	6 (6.3)
Tetralogy of Fallot	5 (5.2)
Partial AV canal defect	2 (2.1)
Total anomalous pulmonary venous drainage	2 (2.1)
Rheumatic valvular heart disease (Pure AR)	2 (2.1)
Quadrileaflet aortic valve with moderate aortic regurgitation	1 (1.0)
Cortriatriatum	2 (2.1)
Valvar Pulmonary stenosis	1 (1.0)
Severe Tricuspid regurgitation	1 (1.0)
Diaphragmatic Paralysis post Fontan	1 (1.0)
Transitional AVSD	2 (2.1)
Rheumatic valvular heart disease (Pure MR)	1 (1.0)
Rheumatic valvular heart disease (Mixed AR and MR)	1(1.0)
Total number of cardiac lesions	96 (100)
RACHS-1, n (%)	
Category 1	26 (35.1)
Category 2	33 (44.6)
Category 3	15 (20.3)
Category 4	0 (0)
Category 5	0 (0)

1 Some cases have combination of cardiac lesions

2 mean (95% confidence interval)

AV= atrioventricular, AVSD=atrioventricular septal defect, RACHS-1= Risk Adjustment for Congenital Heart Surgery-1, AR=Aortic Regurgitation, MR= Mitral Regurgitation

All the patients (except one) underwent their first heart surgery and only 3 (3.9%) of the case had associated syndromes- where 1 (1.3%) case had Down's syndrome and 2 (2.6%) had William's syndrome. There were no laboratory tests done to determine syndromes but rather on general appearance befitting physical descriptions of some syndromes. None had a family history of congenital heart disease. Twenty-nine (47.5%) children had a previous hospital admission documented related to their diagnosed congenital heart disease. Ventricular septal defect (VSD) was identified in 28 (29.5%) cases. Of these, 14 (50%) had peri membranous VSD, 5(18%) has muscular, 5(18%) had peri membranous with an inlet extension 7.7%, 3(10%) subaortic, and the rest had other different types of VSDs. The mean size of the VSD was 11.2 (± 4.7) mm. Patent ductus arteriosus was identified in 32 (33.3 %) cases. Atrial septal defect (ASD) was seen in 10 (10.4%) children with Secundum ASD as the commonest type. The mean size of ASD was 14.1 (±8.1) mm. Severe pulmonary hypertension as per preoperative TTE was identified in 45 (59%) cases whereas mild and moderate pulmonary hypertension was identified in 18 (23%) (Table 1).

A total of 99 procedures were done, with some children requiring more than one procedure during the same surgery. PDA Ligation, VSD repair, ASD Repair, Complete repair of TOF, DSS resection, AV Repair, MV Repair were done in 30 (30%), 27(27%), 14(14%), 5 (5%) and 4 (4%), 4(4%), 2(2%) children, respectively. According to the RACS-1 category, 26 (35.1) were in category 1, 33 (44.6) were in category 2, 15 (20.3) were in category 3 and none of the children were in category 4 and 5 (Table 2).

**Table 2:** Types of cardiac surgery performed at the Children cardiac center, Addis Ababa, Ethiopia

Types of cardiac surgery	N = 99
PDA ligation	30 (30.3)
VSD repair	27 (27.2)
ASD repair	14 (14.1)
TOF complete repair	5 (5)
Aortic valve repair	4 (4)
DSS resection	4 (4)
Partial AV Canal Repair	2 (2)
Transitional AV Canal Repair	2(2)
Membrane Resection for Cortriatriatum	3 (3)
Mitral Valve Repair	2 (2)
Tricuspid Valve Repair	2 (2)
AV repair	1 (1)
TAPVD Repair	2 (2)
Diaphragmatic Plication through Rt Thoracotomy	1 (1)

Abbreviation: AoX time; Aortic clamp time, CPB; Cardiopulmonary bypass, ICU; intensive care unit

Midline sternotomy 53 (70%) was the commonest surgical approach followed by left thoracotomy 23 (30.2%) and only one patient underwent right thoracotomy for plication of postoperative diaphragmatic paralysis after completion of Fontan circulation abroad. Fifty-three (70%) needed CPB. The median CPB and Aox time were 123 and 86 minutes, respectively. Post-operatively, 65 (85.5%) children needed mechanical ventilation only on the day of surgery with the center exercising a rule of early extubation. One patient needed eight days of ventilation due to a complicated ICU course (Table 3).

**Table 3:** Outcomes of cardiac surgery among children operated at the Children cardiac center, Addis Ababa, Ethiopia

Outcomes	N=76
Use of CPB, n (%)	53 (70)
CPB time, median (min), minimal-maximal	123 (41–459)
AoX time, median (min), minimal-maximal	86.5 (21–344)
ICU stay, median (days), minimal-maximal	3 (1–21)
Duration of mechanical ventilation, median (days), minimal-maximal	1 (1–8)
Duration of Ventilation >1 day and <7 days	10 (13.3)
Use of ventilator >7 days, n (%)	1 (1.3)
Hospital Stay, median (days), minimal-maximal	6 (3–35)
Hospital stay ≥14 days, n (%)	2 (2.6)

Abbreviation: AoX time; Aortic clamp time, CPB; Cardiopulmonary bypass, ICU; intensive care unit

Thirteen (17%) children had post-operative complications. Arrythmia 5(6.8%) was the most frequent post-operative complication. Pneumonia 3(4.1%), multiorgan failure 3(3.1%) and open sternum 2(2.7%) were other frequent complications (Table 4).

**Table 4:** Complications after cardiac surgery among children operated at the Children cardiac center, Addis Ababa, Ethiopia^1^ (number of patients = 74)

Complications	Number (%)
Arrythmia	5 (6.8)
Pneumonia	3 (4.1)
Multiorgan failure	3 (4.1)
Open Sternum	2 (2.7)
Chylothorax	1 (1.4)
Renal failure	1 (1.4)
Bleeding requiring reoperation	1 (1.4)
Unplanned cardiac reoperation	1 (1.4)
Pericardial effusion	1 (1.4)
Seizure	1 (1.4)

1 In some cases, there were more than complication per patient.

Post-operative inotropic support was required in 40 (52%) children. Of these, 16 (40%) were taking two or more types of inotropes. Adrenaline was given to 18 (45%) children. Noradrenaline, dobutamine and milrinone was taken by 4 (5.2%), 6(7.8%) and 12(26%) respectively. Post-operative medications given to patients included furosemide (n=58, 95.1%), spironolactone (n=13, 21.3%), enalapril (n=40, 65.6%), sildenafil citrate (n=9, 14.8%), acetylsalicylic acid (n=3, 4.9%), captopril (n=1, 1.6%) and atenolol (n=1, 1.6%). In a univariate analysis, need for inotrope (OR=6.1; 95%CI1.5, 25.1), days on mechanical ventilator (OR=5.5; 95%CI1.5, 19.8) and length of ICU stay (OR=2.0; 95%CI1.0, 3.9) were significantly associated with the presence of complications after cardiac surgery. However, in final multivariate model, none showed significant association with complications (Table 5).

**Table 5:** Correlates of complications after cardiac surgery among children operated at the Children cardiac center, Addis Ababa, Ethiopia

Correlates	Complications		Unadjusted Odds Ratio (95% CI)
	
	No	Yes	
Previous hospital admission, n (%)	7 (19.4)	7 (20.0)	1.0 (0.3, 3.2)
Need for inotrope support, n (%)	20 (62.5)	12 (37.5)	4.9 (1.4, 17.2)*
Days on mechanical ventilator, mean (SD)	1.1 (0.3)	1.9 (1.8)	5.5 (1.6, 19.2)*
Duration of CPB, median [IQR]	123 [100–150]	131 [108–220]	1.0 (0.99, 1.02)
Length of ICU stay, median [IQR]	3 [2–4]	4 [3–5]	2.1 (1.1, 3.9)*

* P<0.05; CPB= Cardiopulmonary bypass, ICU= intensive care unit, IQR=interquartile range, SD= standard deviation

The median length of intensive care unit and hospital stay were 3 and 6 days respectively. Fifty-nine (97.4%) were alive at discharge. In-hospital mortality was 2.6% and there was no patient who died within 30 days after discharge. One 6 years old girl who developed and treated for Post cardiotomy syndrome 5 weeks after her first surgery died 3 months into her follow up as a result of a home cardiac arrest that was not resuscitated effectively. Operative mortality was 2.6% the mortality summary is reported (Table 6).

**Table 6:** Summary of moralities after cardiac surgery among children operated at the Children cardiac center, Addis Ababa, Ethiopia

Diagnosis	Age (months)	Surgical procedure	Cause of death
TOF + Quadrileaflet AV with Moderate AR + Multiple Tet Spells	60	Complete TOF Repair (TAP) + AV Repair	MOF
TOF + Multiple Tet Spells	24	Complete TOF Repair (Valve Sparing)	MOF

AV=Aortic Valve, MOF=Multiple Organ Failure, TAP=Trans annular patch, TOF=Tetralogy of Fallot

## Discussion

To our knowledge this is the first study assessing the outcomes and correlates of complication of pediatric cardiac surgery in Ethiopia. Most patients had acyanotic congenital heart disease. Post-operative complications are associated with patient morbidity, mortality and increase hospital stay (13). Studies from Indonesia and Guatemala have reported one-fifth of children who underwent cardiac surgery developed postoperative complications (14). At the Uganda heart institute, complications were identified in 34.3% (15). In our study, the commonest complication reported was arrythmia, which is similar to the study from Uganda (15). Low cardiac output syndrome followed by arrythmia was the commonest complications reported from Indonesia(14).

The 2.6% mortality rate reported in this study is encouraging and are in line with outcomes in other developing countries that reported mortality rates ranging from 0–26.4%[13–15]. A study from Indonesia reported 26.4% operative mortality rate (14) which is higher as compared to our study (3.3%). A single-center study from Uganda reported similar morality rate as our study (15). A small (n=36 cases) single center study from China reported no death after percutaneous interventions for pediatric congenital heart disease s(16) .In a mixed population of acquired and congenital heart diseases, a team from Tanzania reported a a mortality rate of 13.3% out of the 105 patients they operated (17). The Risk Adjustment for Congenital Heart Surgery-1 is a consensus-based tool used to predict in-hospital mortality in children who undergo cardiac surgery (18). Many of our patients have low RACS-1 score making the likelihood of complications and death to be low as compared to open cardiac surgery, percutaneous interventions are known to decrease hospital stay which could potentially reduce post-operative complications and possibly mortality. This could explain the lack of mortality in the Chinese study. Low mortality in our study can be explained by a careful selection of cases to be operated in a low resource set up.

We have shown that the need for post-operative inotrope, number of days on mechanical ventilation and length of ICU stay were associated with presence of complications. Similar findings were identified in the study from Indonesia (13). The need for inotropes could represent low cardiac output that could increase complications. As the days on mechanical ventilation and ICU stay increase the risk of complications such as ventilator associated pneumonia and sepsis could increase (20).

In low resource settings, the diagnosis of congenital heart disease is expected to increase with improving health seeking behavior and cardiovascular investigation. Our center is the only center that can do open-heart surgery on a regular basis for about 110 million population of which more than 40% are under 15. This warrants the establishment of more centers as well as increase the current capacity of our center (currently only works in one-third of its capacity) to treat more patients. With more professionals trained in the field, more hospitals with ability to do open heart surgery and catheterization and better availability of consumables we can see that indeed saving as many children with heart disease is a possibility. This study is intended to show the current realities of pediatric cardiac surgery in Ethiopia. Finally, if more resources are invested to train health professionals and improve the health system as well as purchase of necessary consumables by the government to match the demand the care of congenital heart disease can be enhanced in Ethiopia.

In conclusion, most of the operated patients had acyanotic congenital heart disease. In-hospital and operative mortality was low and encouraging. Further expansion of cardiac surgery service in Ethiopia is recommended.
